# Successful management of a delayed presentation of traumatic descending thoracic aorta pseudoaneurysm: a literature review based on a case report

**DOI:** 10.1186/s12245-024-00670-w

**Published:** 2024-07-15

**Authors:** Mohammad Sadeghian, Pouya Ebrahimi, Parnian Soltani, Massoud Ghasemi, Homa Taheri, Maryam Mehrpooya

**Affiliations:** 1 Department of Cardiology, Tehran Heart Center, Tehran University of Medical Science, Tehran, Iran; 2 Tehran Heart Center, Cardiovascular Diseases Research Institute, Tehran University of Medical Sciences, Tehran, Iran; 3 Department of Interventional Cardiology, Imam Khomeini Hospital Complex, Tehran University of Medical Science, Tehran, Iran; 4 Cedars-Sinai Medical Center, Los Angeles, CA USA; 5 Department of Cardiology, Imam Khomeini Hospital, Tehran University of Medical Sciences, Tehran, Iran

**Keywords:** Transthoracic echocardiography, Non-invasive cardiovascular imaging, Aortic pseudoaneurysm, Interventional cardiology, Endovascular repair, Cardiac trauma

## Abstract

**Background:**

Blunt traumatic aortic injury (BTAI) is the second leading cause of death due to traumas in young patients. The primary presentation might be chest or interscapular pain, difficulty in breathing, and, in severe cases, hypotension. Considering the rapid deterioration of these patients’ clinical conditions, prompt diagnosis and treatment initiation are crucial. In these injuries, the most involved parts of the aorta are the isthmus (distal to the left subclavian artery) and the descending part in the thorax. Therefore, the main diagnostic strategies include transthoracic echocardiography, CT angiography, and endovascular diagnostic approaches.

Case presentation

The patient was a 19-year-old male presenting with the symptoms of chest pain, dyspnea, and extremities excruciating pain after a car turnover. The initial evaluation showed no abnormal cardiovascular finding except bilateral hemothorax, addressed with chest tubes. Twelve hours later, when the patient was under observation for orthopedic surgeries, his chest pain and dyspnea started, and TTE and CTA showed a grade three descending aneurysm of the aorta. The patient was treated immediately with an endovascular procedure of stent implantation. A delayed debranching surgery was also performed, which resulted in desirable outcomes and uneventful follow-up.

**Conclusion:**

Although open thoracic surgery is the main and almost the only option for treating aneurysms of the aorta in hemodynamically unstable patients, the endovascular procedure has shown superior outcomes in selected patients with appropriate anatomy. Debranching surgery, which can be done simultaneously or with delay after the initial procedure, has proven protective against thromboembolic cerebral events.

**Clinical key point:**

Patients with an aneurysm of the aorta should be transported to a medical center with a multidisciplinary team for an urgent evaluation and treatment. The initial resuscitation and diagnosis are challenging, considering the fatal nature of these injuries, and the selection of the treatment is based on the patient's clinical condition and evaluated anatomy in cardiovascular imaging.

## Introduction

The leading cause of death in young adults is trauma, and the main consequences that result in mortality are head injuries followed by blunt thoracic aortic injuries (BTAI) [1]. The main mechanisms that can cause these injuries include motor-vehicle crashes, pedestrian vs. motor-vehicle collisions, falls from a considerable height, and crush injuries [1]. An extremely high risk of mortality accompanies BTAI if they are not diagnosed and addressed immediately [2]. It has been concluded from previous surveys about four-fifths of patients with BTAI die before being transported to the hospital, and those who reach the medical center have a mortality of 46% [3–5]. The most commonly involved section of the aorta is its isthmus (exactly distal to the subclavian artery). The reason for such a vulnerability is determined to be its location between the more mobile part (ascending aorta and arch) and the relatively fixed zone of the descending thoracic aorta [6]. The entrapment of the isthmus section between anterior and posterior bony structures also makes this part more vulnerable to focal rupture [6]. Moreover, the aortic tissue in the isthmus has weaker tensile, making it intrinsically more prone to these injuries [7, 8].

The initial presentation of BTAI varies based on the mechanism and the severity of the injury, ranging from asymptomatic cases with incidental findings on images to more severe mechanisms, which are brought to the hospital complaining of chest or interscapular pain, difficulty in breathing, or symptoms of hypotension such as fainting, dizziness, or in severe cases even declined level of consciousness (in 40% of cases) [9].

This study presents the history and clinical progression of a 19-year-old male patient with severe descending BTAI and a delayed presentation of the descending aorta pseudoaneurysm, managed successfully by an endovascular approach after the initial resuscitation.

## Case presentation:

### *Patient’s history and* physical examination

He patient was a 19-year-old male who was brought to the emergency room due to complaining of severe pain in his lower back, upper and lower limbs, and also in his genital area after having a severe motor vehicle (car turnover) accident. He was driving a car at high speed when he confronted another car, and they collided, which resulted in the deviation and turnover of the car. In his initial general appearance evaluation, he was conscious, anxious, and moaning due to pain all over his body, especially in his extremities. He could not move the lower part of his body (below his pelvis) due to severe pain and was immobile on the couch. In his physical exam, the vital signs were stable, except for tachycardia (Heart rate:130, Blood pressure: 110/70 mmHg, Respiratory rate: 19, Temperature: 36.5, and oxygen saturation: 96% on ambient air). There were several lacerations with a superficial appearance and no bleeding on his skin. His left forearm and ankle deformity were also obvious. Upon his head, neck, and nervous system examination, no remarkable sign of brain injury or intracranial bleeding could be detected. Cardiovascular and neurologic examinations were normal. The movement of the chest was restricted due to the severe pain in the chest during respiration. Reduced air sounds in auscultation and dullness in percussion were detected on the right chest, suggesting hemothorax. Therefore, a chest tube was inserted urgently after the confirmation by an immediate, portable chest X-ray in the emergency room. Abdominal examination was unremarkable. An excruciating tenderness in his pelvis and lower limb, especially the left ankle, was also obvious.

## Methods

**Fig. 1:**
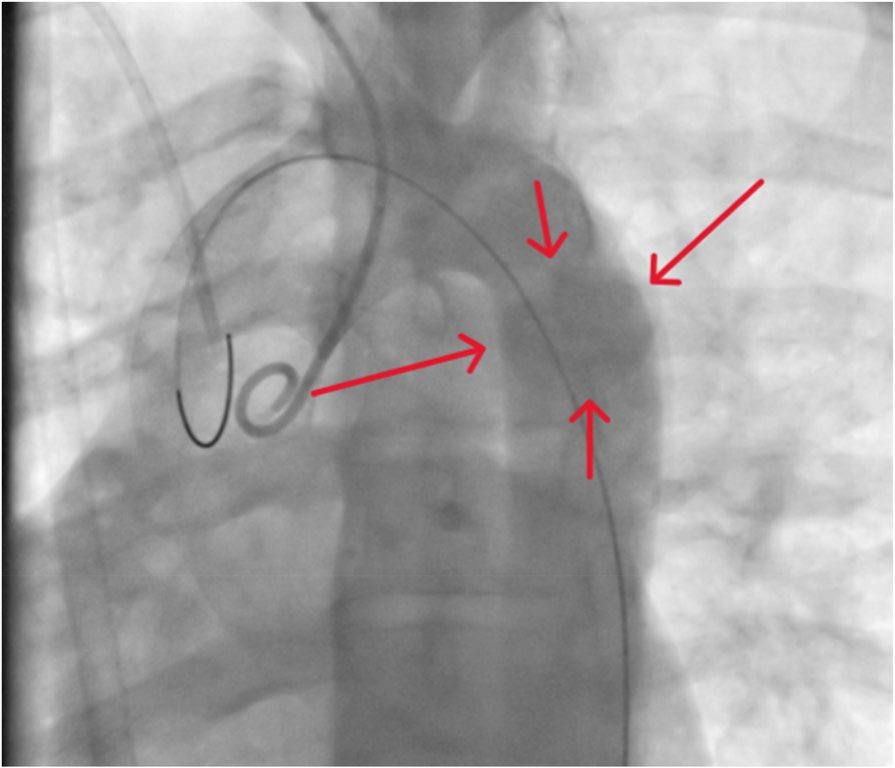
Aortic angiography and the leak of contrast showing pseudoaneurysm (red arrows)

The resuscitation with intravenous (IV) fluid (Normal Saline 0.9%) and analgesics (Morphine Sulfate 3mg) was initiated, and at the same time, focused assessment with sonography for trauma (FAST) was performed and revealed no sign of internal bleeding. In the imaging evaluations, including brain, chest, abdomen, and pelvic computed tomography (CT) scan, the remarkable observation was the bilateral sacral, left hand, and ankle fractures. No remarkable abnormality of the aorta was seen in the initial radiologic evaluation. Conservative management for the stabilization of the patient for his orthopedic surgeries was initiated, and he was transferred to the intensive care unit (ICU). The day after the surgery, the patient complained of severe chest pain and was breathless with efforts. After providing oxygen (5 Lit/Min) and obtaining an electrocardiogram (ECG), which was normal, the medical team repeated his chest X-ray. The portable chest x-ray revealed mild widening of the mediastinum in this patient. This made the diagnosis of an aneurysm of the thoracic aorta the main differential diagnosis. Therefore, an urgent transesophageal echocardiogram was requested, which confirmed the diagnosis of a grade III (pseudoaneurysm) aortic aneurysm. After the resuscitation and stabilizing the patient's clinical condition, an immediate chest CT angiography was conducted, showing the outpouching of dye in the initial part of the descending thoracic aorta and mediastinal fat dirtiness**,** suggestive of descending aorta pseudoaneurysm. A bedside transthoracic echocardiogram showed a haziness in the proximal part of the descending aorta, mild left and right ventricle enlargement, diastolic dysfunction, inferior vena cava plethora, high pulmonary arterial pressure (systolic pulmonary arterial pressure 30-35 mmHg). An angiography was scheduled for additional information and to plan the endovascular procedure. Arterial access from the right radial artery was obtained, and a 6-french sheath was used. Aortography was done with precaution, and a sign of contrast extravasation in a pouch was compatible with pseudoaneurysm (Fig. 1)*.*

**Fig. 2:**
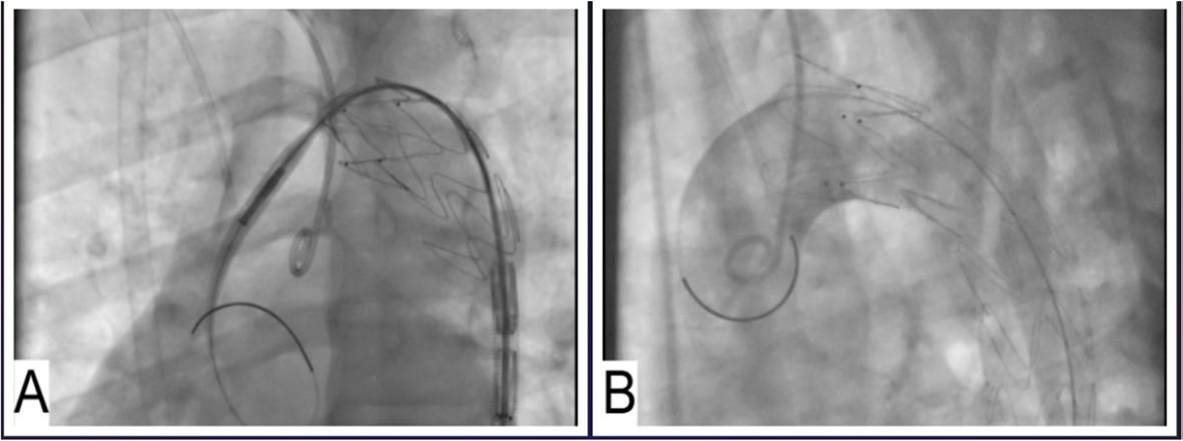
Contrast angiography during (**A**) and after (**B**) the implantation of a Zenith alpha stent

Endovascular intervention was successfully performed by stenting the thoracic aorta just after the left common carotid artery with the implantation of a Zenith alpha stent 26*105 (Cook Medical Company)***.*** The final injection and follow-up CT angiography showed the patency of the left carotid and subclavian artery (Fig. 2). The patient’s chest pain and respiratory symptoms improved dramatically. Debranching was supposed to be performed early after the procedure. Surgical debranching of the left subclavian artery was done to occlude the ostium of the left subclavian artery.

### Outcome and follow-ups

**Fig. 3:**
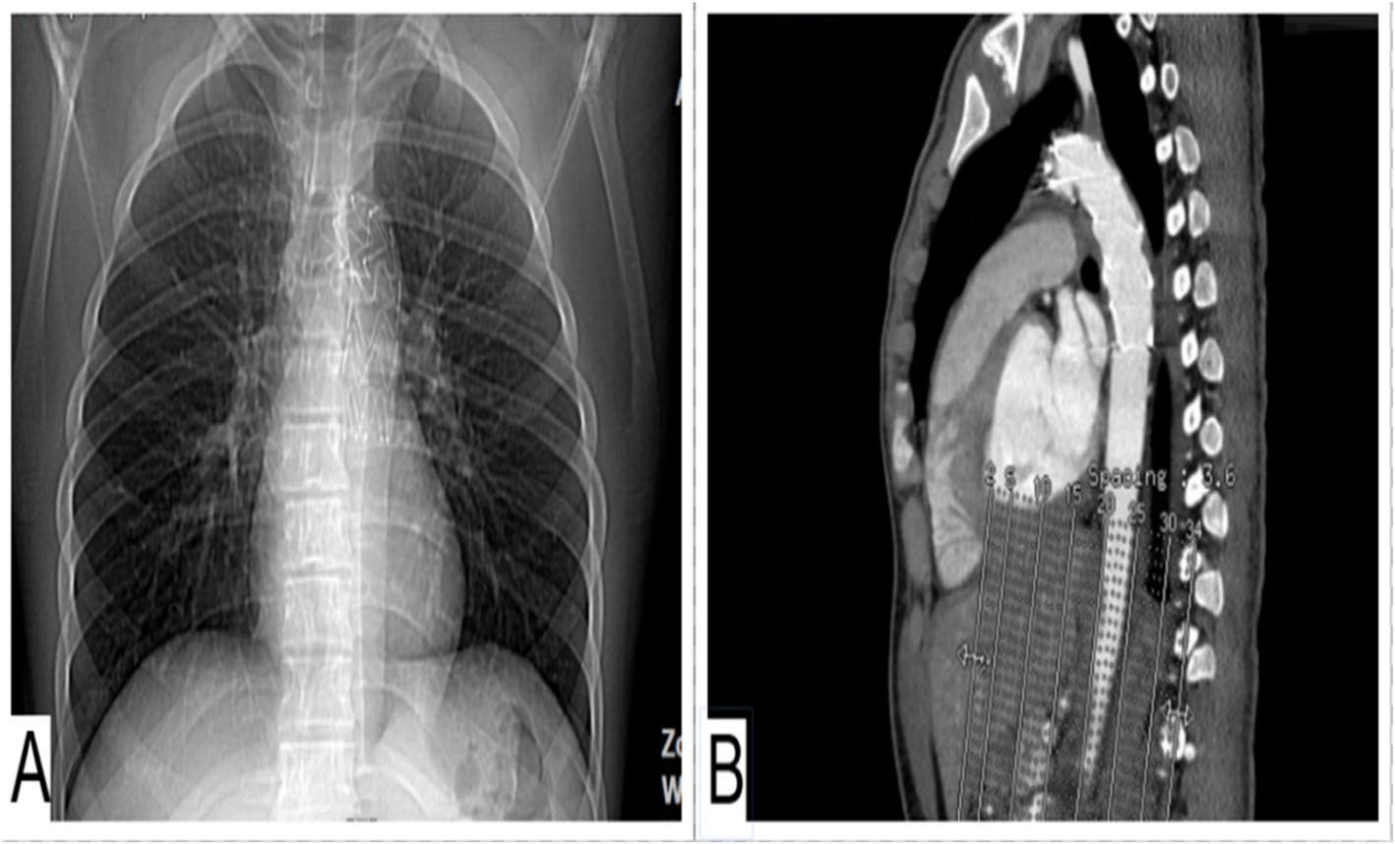
Follow-up Chest X-ray (**A**) and CT angiography (**B**) 45 days after the implantation of the stent and debranching surgery

He was discharged a week after the debranching surgery without any remarkable symptoms and with an appropriate general condition. His one-year follow-up was uneventful, and all his laboratory and imaging (Fig. 3)*.* were unremarkable, with no signs of endoleak or other problems.

## Discussion

In this case report and literature review, the history and progression of a 19-year-old man with a pseudoaneurysm of descending aorta due to BTAI were presented. The patient was initially stabilized, and no sign of aortic injury was seen in the first presentation. However, after 12 hours, the patient’s condition deteriorated. However, the pseudoaneurysm was treated by inserting a stent into a thoracic endovascular aortic repair (TEVAR) procedure.

### Diagnosis

Symptoms and signs of aortic aneurysm are not specific. Therefore, the diagnosis of this potentially fatal condition at an appropriate time is not always straightforward. On the other hand, early diagnosis, especially in severe cases, has been associated with a significant reduction in the risk of morbidity and mortality [10]. The initial evaluation almost always begins with an anteroposterior chest X-ray. Although it has insufficient sensitivity and specificity, some hints can be obtained for the diagnosis, from the most common ones, such as the widening of the mediastinum, to less prevalent ones, including loss of aortic knob, apical capping from blood in the apex, large left pleural effusion (hemothorax), displacement of left mainstem bronchus, deviation of an oral or nasogastric tube to the right, deviation of the trachea or right mainstem bronchus and widening of the paravertebral stripe [1]. In the emergency departments, a Focused assessment with sonography for trauma (FAST) can be useful in severe cases where the rupture can lead to hemothorax or pericardial effusions. However, this method also has low sensitivity and specificity [1, 11].

When the diagnosis of BTAI is suspected, the confirmation or ruling out needs more sensitivity and specificity. Transesophageal echocardiograms (TEE), along with contrast-enhanced CT-scan, have been the mainstay of the diagnosis [12]. The advantage of echocardiograms over CT scans is less radiation exposure. The other factor that should be considered for the imaging modality selection is the patient’s clinical condition. For those with unstable hemodynamic conditions, a prompt bedside TEE is preferred, while stable patients are mostly transferred to do the contrast-enhanced CT scan [13]. The accuracy of TEE and CECT is almost the same in the detection of early subadventitial aortic injuries. In contrast, For a BTAI, CECT has a distinguished sensitivity (95-100%) and almost perfect negative predictive value (100%) [14]. The other advantage of CECT over the TEE is providing the ability to extend the evaluation from the ascending part to the descending thoracic and abdominal aorta. The presence of intimal damages, from the flap to complete rupture of the vessel wall (contained or total rupture), the observation of luminal filling defects, and the detection of aortic contour abnormalities such as periaortic hematoma or pseudoaneurysm are the main indicators of aortic trauma which are seen in CECT [14].

Despite the aforementioned advantages of CECT, patients who are not stable enough to be transferred to the CT department would benefit from the diagnostic accuracy of bedside TEE, such as what happened to the patient presented in this study [15]. If an expert echocardiographer performs the procedure, it can have almost 90-100% sensitivity and almost 98-100% specificity for the diagnosis of the BTAI. The average precision is even higher in cases of ascending or isthmus BTAI [16]. The findings of BTAI in TEE are irregular intraluminal flaps transversing the lumen of the aortic isthmus, which can be seen in the TEE transverse view. Furthermore, the pseudoaneurysm might be detected as the presence of an abnormal aortic contour [15]. Wall disruption of the aorta, which leads to local blood flow turbulence or the obstruction of the aorta caused by pseudo-coarctation, can be found by adding the color Doppler and continuous wave Doppler assessment [15]. The determination of the severity of the injury would help plan the therapeutic steps. The differentiation of minimal aortic injury (MAI) and significant aortic injury (SAI) is mainly performed based on the absence or presence of external aortic wall deformity according to the Society of Vascular Surgery classification [17]. There are four types of MAI and SAI, ranging from an initial tear or flap (Type I) to an open rupture (Type IV). Grades II and III are defined as intramural hematoma and pseudoaneurysm, respectively [18].

### Management

The severity of the BTAI and clinical circumstances are the main determining factors for determining this treatment's management steps, ranging from conservative management in the first-grade trauma to emergent surgery in the complete rupture. In cases with grades two and three and the presence of appropriate anatomy, management can be planned with delayed TEVAR. [19].

The most crucial action in the initial management of BTAI patients is moving forward step by step based on Acute Traumatic Life Support (ATLS) guidelines and stabilizing the vital signs. Another important point is the availability of an expert multidisciplinary team, including a general surgeon, an emergency specialist, an orthopedic surgeon, a critical care and anesthesia specialist, and trained nurses and paramedics. In insufficient logistic facilities, the patient should be stabilized and transferred to an appropriately prepared facility by medical escort. In the stabilization process, the patient’s airway should be evaluated with spine precautions (Airway), and supplementary oxygen should be provided to reach a blood oxygen saturation of 94-98%. Drainage should be conducted immediately in cases of tension pneumothorax (Breathing) [20].

Maintaining the appropriate blood circulation is the next step, as is controlling any source of bleeding in addition to the IV transfusion of fluid through one or two confident IV access, especially in cases with systolic blood pressure less than 90mmHg or a heart rate ≥120 per minute. Calcium chloride infusion (with a dose of 0.2 mL/kg and 1000 mg maximum) is highly recommended in cases suspected of hemorrhagic shock (Circulation). The temperature of the patient’s body should be managed carefully, and in cases of hypothermia, removing wet clothes, providing a blanket, or more invasive warming procedures based on the severity of the condition are required. Finally, any other probable injury, specifically brain injuries and hemorrhages, as the leading cause of death due to trauma, should be evaluated (Disabilities) [20–23].

In the past three decades, endovascular procedures have widely replaced open surgeries, and in the cases of BTAI, the success rate of these procedures is reported as high as 80-100%. Despite widely different outcomes published regarding the result of the TEVAR procedure in BTAI, they mostly supported this type of treatment as an alternative to open thoracic surgeries. They demonstrated an improved outcome. [24, 25]. Therefore, this procedure is considered the first-line therapy for patients with favorable anatomic structures. Some studies have shown an improvement in these patients' morbidity and mortality with early endovascular repair application [26].

Compared to open surgical repair, taking advantage of endovascular approaches like TEVAR has demonstrated a considerable decline in morbidity and mortality in these injuries. Two of the most important applications of TEVAR are pseudoaneurysms, aneurysms, and type B dissection of the aorta [27, 28]. Although it has been stated that the early initiation of the process has been associated with lower mortality and morbidity, the procedure can be performed with a planned delay in patients with concomitant traumas and the need for stabilization [29]. An important point that should be considered in the planning is stent graft devices might not be appropriate for implantation in the narrow and tight angulation of the aortic arch in 20-30% of cases. Therefore, by considering all these details, selecting the stent and the patient’s plan would increase the chance of achieving the optimal outcome [30].

One of the principles of ensuring appropriate coverage of the aorta is the coverage of the left subclavian artery. Any ischemic neurovascular injury should be prevented in these cases, and a bypassing procedure (debranching) should be performed. Previous studies have demonstrated a significant increase in the risk of neurovascular complications in cases with the coverage of the left subclavian and without a debranching procedure [2, 31].

In the presented case, the patient was successfully resuscitated and stabilized. The delayed presentation of the aorta pseudoaneurysm (grade three of aortic injury) was managed with a TEVAR procedure and then a debranching surgery to prevent ischemic accidents of the brain. Choosing TEVAR (instead of open surgery) resulted in the desired outcome. It could save the patient's life without considerable short- and long-term complications related to this procedure. The extent of concomitant injuries and the critical condition of the patient strongly restricted us from performing the debranching process before TEVAR. Moreover, the orthopedic complications caused septic arthritis and resultant sepsis. Therefore, debranching could not be done on index admission. This procedure was completed when the patients became hemodynamically stable and free from infection and, thus, survived severe complications of the injury.

### Clinical key point (take-home message)

BTAI is a potentially rapidly fatal condition with an unspecific presentation, and the lack of sensitivity in initial diagnostic strategies makes diagnosis challenging. TTE and CECT are the mainstay of diagnosis in stable and unstable patients, respectively. On-time diagnosis and treatment crucially need a multidisciplinary approach. Immediate resuscitation based on the ATLS guidelines (A: Airway, B: Breathing, C: Circulation, D: Disabilities) and surgical or endovascular repair initiation can save these patients' lives. Careful planning for the follow-up of these patients is also crucial. The debranching surgery can be performed in the same index intervention or a delayed procedure, depending on the patient's clinical condition.

## Data Availability

Data is available on request due to privacy/ethical restrictions.
